# ISG15 targets glycosylated PD-L1 and promotes its degradation to enhance antitumor immune effects in lung adenocarcinoma

**DOI:** 10.1186/s12967-023-04135-1

**Published:** 2023-05-22

**Authors:** Tongyuan Qu, Wenshuai Zhang, Chenhui Yan, Danyang Ren, Yalei Wang, Yuhong Guo, Qianru Guo, Jinpeng Wang, Liren Liu, Lei Han, Lingmei Li, Qiujuan Huang, Lu Cao, Zhaoxiang Ye, Bin Zhang, Qiang Zhao, Wenfeng Cao

**Affiliations:** 1 Department of Pathology, Tianjin Medical University Cancer Institute and Hospital, National Clinical Research Center for Cancer, Tianjin’s Clinical Research Center for Cancer, Tianjin, China; 2 Department of Pediatric Oncology, Tianjin Medical University Cancer Institute and Hospital, National Clinical Research Center for Cancer, Tianjin’s Clinical Research Center for Cancer, Tianjin, China; 3 Department of Breast Cancer, Tianjin Medical University Cancer Institute and Hospital, National Clinical Research Center for CancerKey Laboratory of Breast Cancer Prevention and Therapy, Tianjin Medical University, Ministry of EducationTianjin’s Clinical Research Center for Cancer, Tianjin, China; 4 Department of Gastrointestinal Cancer Biology, Tianjin Medical University Cancer Institute and Hospital, National Clinical Research Center for Cancer, Tianjin’s Clinical Research Center for Cancer, Tianjin, China; 5 Cancer Molecular Diagnostics Core, Tianjin Medical University Cancer Institute and Hospital, National Clinical Research Center for Cancer, Tianjin’s Clinical Research Center for Cancer, Tianjin, China; 6 Department of Radiology, Tianjin Medical University Cancer Institute and Hospital, National Clinical Research Center for Cancer, Tianjin’s Clinical Research Center for Cancer, Tianjin, China

**Keywords:** ISG15/ISGylation, Glycosylated PD-L1, Anticancer immunity, Lung adenocarcinoma

## Abstract

**Background:**

Immunocheckpoint inhibitors (ICIs) have been widely used in the clinical treatment of lung cancer. Although clinical studies and trials have shown that patients can benefit significantly after PD-1/PD-L1 blocking therapy, less than 20% of patients can benefit from ICIs therapy due to tumor heterogeneity and the complexity of immune microenvironment. Several recent studies have explored the immunosuppression of PD-L1 expression and activity by post-translational regulation. Our published articles demonstrate that ISG15 inhibits lung adenocarcinoma progression. Whether ISG15 can enhance the efficacy of ICIs by modulating PD-L1 remains unknown.

**Methods:**

The relationship between ISG15 and lymphocyte infiltration was identified by IHC. The effects of ISG15 on tumor cells and T lymphocytes were assessed using RT-qPCR and Western Blot and in vivo experiments. The underlying mechanism of PD-L1 post-translational modification by ISG15 was revealed by Western blot, RT-qPCR, flow cytometry, and Co-IP. Finally, we performed validation in C57 mice as well as in lung adenocarcinoma tissues.

**Results:**

ISG15 promotes the infiltration of CD4^+^ T lymphocytes. In vivo and in vitro experiments demonstrated that ISG15 induces CD4^+^ T cell proliferation and invalidity and immune responses against tumors. Mechanistically, we demonstrated that the ubiquitination-like modifying effect of ISG15 on PD-L1 increased the modification of K48-linked ubiquitin chains thus increasing the degradation rate of glycosylated PD-L1 targeting proteasomal pathway. The expression of ISG15 and PD-L1 was negatively correlated in NSCLC tissues. In addition, reduced accumulation of PD-L1 by ISG15 in mice also increased splenic lymphocyte infiltration as well as promoted cytotoxic T cell infiltration in the tumor microenvironment, thereby enhancing anti-tumor immunity.

**Conclusions:**

The ubiquitination modification of PD-L1 by ISG15 increases K48-linked ubiquitin chain modification, thereby increasing the degradation rate of glycosylated PD-L1-targeted proteasome pathway. More importantly, ISG15 enhanced the sensitivity to immunosuppressive therapy. Our study shows that ISG15, as a post-translational modifier of PD-L1, reduces the stability of PD-L1 and may be a potential therapeutic target for cancer immunotherapy.

**Supplementary Information:**

The online version contains supplementary material available at 10.1186/s12967-023-04135-1.

## Introduction

According to the latest statistics, more than two million new lung cancer patients are diagnosed each year worldwide, making it a significant cause of cancer-related deaths worldwide. Over 85% of these are currently classified as non-small cell lung cancer (NSCLC). Survival rates for NSCLC patients have not improved significantly, with a proportion of NSCLC patients being diagnosed at an intermediate to advanced stage; another proportion developing local or systemic metastatic disease leading to a poorer prognosis for these patients, with an overall 5-year survival rate of less than 20% [1–3]. In addition to surgery and conventional chemoradiotherapy, Immune checkpoint inhibitors (ICI) have emerged in the treatment of lung cancer in recent years. Aiming at Programmed cell death-1 (PD-1) and Programmed cell death-ligand 1(PD-L1) mab has been widely used in the clinical treatment of non-small cell lung cancer (NSCLC), but tumor heterogeneity and the complexity of immune microenvironment result in less than 20% of patients benefiting from ICI therapy [4]. These patients are also prone to drug resistance and relapse [5–7]. PD-L1 protein expression changes dynamically in the process of tumor progression, with a low positive staining rate, and immunotherapy is affected by several factors, which require us to conduct in-depth studies on the regulation mechanism of PD-L1 protein expression to improve the benefit of PD-L1 monotherapy [8].

Loss or mutation of IFN signaling pathway function is associated with immune escape to resistance to ICI [9]. Interferon-stimulated gene 15 (ISG15) is one of the key downstream molecules of the IFN-I pathway. ISG15 shares structural and mechanistic features with ubiquitin. ISGylation occurs similar to the enzymatic cascade reaction of ubiquitin, exposing the glycine sequence at the C-terminus of ISG15 in the presence of a ligase and linking it to the lysine of the target protein. ISG15 is present in the cell in unconjugated and conjugated form and is secreted extracellularly in its free form [10–12]. Several studies have confirmed the dysregulation of ISG15 and ISGylation expression in tumours [13–15]. Targeting ISG15 to treat tumours appears to be a promising strategy. In our published article, we have elucidated that ISG15 acts as an independent prognostic marker for lung adenocarcinoma (LUAD) and undergoes ISGylation with ESRP1, reducing ESRP1 degradation. At the same time, ESRP1 transcriptionally regulates ISG15 and the two form a positive feedback loop that together inhibit LUAD progression [16].

PD-L1 is mediated by multiple post-translational modifications (PTMs) including but not limited to glycosylation, ubiquitination, phosphorylation and palmitoylation [17–19]. Targeting glycosylated PD-L1 has made significant advances in activating anti-tumour immunity. Whether ISG15, as a post-translational modifier, can target post-translational modifications of PD-L1 remains to be explored.

In the present study, we will focus on the ubiquitination modification of glycosylated PD-L1 by ISG15. We identified PD-L1 can serve as a new target protein for ISG15, thus enabling ISG15 to perform ubiquitination modification on glycosylated PD-L1 to promote its degradation and thus reduce the expression of PD-L1. We highlighted the significance of ISG15 in LUAD therapy and we clarify the possibility of ISG15 involvement in the benefit of ICI therapy, which is expected to provide new therapeutic ideas and strategies for the immunotherapy of LUAD.

## Materials and methods

### Clinical samples

A total of 153 lung adenocarcinoma specimens were collected in 2012 at the Department of Pathology, Tianjin Medical University Cancer Hospital. One specimen was obtained from each patient and each specimen was diagnosed as primary LUAD by two pathologists. All specimens were obtained before the patients received chemotherapy and radiotherapy. Clinical and pathological information on the patients was complete, and follow-up information was available until December 2020. Our study protocol in the study were approved by the Hospital Human Subjects Protection Committee. This study was approved by the Hospital Human Subjects Protection Committee.

### Cell culture

The human LUAD cell lines A549, H1299 and Pc-9 and the Lewis mouse lung cancer cell line (LLC) were purchased from the Shanghai Type Culture Bank of the Chinese Academy of Sciences. A549, H1299 and PC9 cells were grown in RPMI-1640 (Gibco) medium supplemented with 10% fetal bovine serum (FBS, Pricella, 164210-50), 1% penicillin–streptomycin (Pricella, PB180120). LLC cells were cultured in 10% fetal bovine serum (FBS, Pricella, 164210-50), 1% penicillin–streptomycin (Pricella, PB180120) in DMEM (Gibco) medium. In 5% CO_2_ humidified atmosphere, cell cultures were maintained at 37 °C. Experimental cells were in the logarithmic growth phase.

### Lentiviral infection and plasmid transfection

Human and mouse ISG15 overexpression and human ISG15 knockdown lentivirus and USP18 plasmids were purchased from Genechem (Shanghai, China). LUAD cells of logarithmic growth phase were transfected with lentivirus and subsequently screened by adding puromycin (2 μg/μl). Transfections were performed with LipoFiter (Hanbio, China) according to the manufacturer's instructions. Expression of ISG15 and USP18 was assessed by Western blotting and RT-qPCR.

### Antibodies and reagents

Antibody information and dilutions are described in Additional file 6: S6.

### Real-time PCR

An extraction of total RNA from cells was performed using TRIzol. The RNA was then reverse transcribed to cDNA using the StarScriptiiiRT kit (A232, GenStar, China). Quantitative reverse transcription-PCR (RT-qPCR) was performed using RealStar Power SYBR qPCR Mix (A311, GenStar, Chian) as manufacturer’s recommended protocol. The target gene primers were as follows:

ISG15 (forward5′-TGGACAAATGCGACGAACCTC-3′;

Reverse5′-TCAGCCGTACCTCGTAGGTG-3′),

Actin (forward5′-CGAGATCCCTCCAAAATCAA-3′;

Reverse 5′-CGAGATCCCTCCAAAATCAA-3′),

PD-L1 (forward5′-TGCCGACTACAAGCGAATTACTG-3′;

Reverse 5′-CTGCTTGTCCAGATGACTTCGG-3′).

### Co-culture assays

CD4^+^T cells were isolated from peripheral blood obtained from a healthy donor with the use of a CD4^+^ T-cell isolation kit (Miltenyi Biotec). After 24 h activation with 2 µg/ml CD3 antibody and 1 µg/ml CD28 antibody (BioLegend), activated CD4^+^ T cells were co-cultured with 10 Gy-irradiated A549/H1299 cells in a 5:1 ratio. Tumour cells were collected and Western blot was performed to detect apoptotic proteins after 48 h of culture. Using CCK-8 assay (Elabscience Biotechnology Co), CD4^+^ T cells were evaluated for proliferation. The supernatants were harvested after 48 h and IFN-γ and ISG15 secretion were determined by ELISA (eBioscience).

### Immunoprecipitation

Immunoprecipitation analysis of ISG15 and PD-L1 was performed using an immunoprecipitation kit (Sangon Biotech, Chian, No. C600689). 4 µg of Anti-ISG15 antibody or anti-IgG antibody was added to the cell lysates and incubated for 12 h at 4 °C. The anti-PD-L1 antibody was used to probe the immunoprecipitate proteins after their elution with SDS-PAGE sample buffer and resolving by SDS-PAGE.

### Protein half-life assays

Lung adenocarcinoma cells were treated with 20 μM cycloheximide (CHX) or DMOS (Control), respectively, and protein was collected at different time points. Changes in PD-L1 protein levels were next detected using Western blot and quantified using Image J.

### Animal models

The C57BL/6 female mice were obtained from Si Bei Fu Biotechnology (Beijing, China) at 5 weeks of age. The mice were randomly grouped in groups of five mice and each mouse was injected with 1 × 10^6^ LLC mouse lung cancer cells (stable clones overexpressing ISG15, and WT). Tumour volume = (width^2^ × length)/2 according to the formula, and the length and width of tumours in mice were observed every 2 days. In the antibody-based pharmacological intervention, we administered 100 mg of anti-PD-L1 antibody (A2004, Selleck) or solvent control intraperitoneally every 3 days, respectively. The end point of the animal experiment was that the mice were killed by cervical dislocation when the xenograft volume reached 1500 mm^3^ or when ulcers developed. Spleen, lung, and tumor tissues were analyzed by HE, Immunohistochemistry (IHC), and flow cytometry. The Animal Experimentation Ethics Committee of Tianjin Medical University Cancer Hospital and Research Institute approved all animal experiments in this study.

### Flow cytometry analysis

To generate single-cell suspensions, the tumors and spleen tissues were cut into small pieces, mechanically disrupted, and filtered through a 70-μm strainer. Subsequently, the filtered single cell suspension was mixed with red blood cell lysate (BD Biosciences) to lyse the intermingled red blood cell. Finally, single cell suspensions were combined with fluorescein-labeled antibodies for 1 h at room temperature. The centrifuged single cells were resuspended in PBS and analysed using a flow cytometer (BD Biosciences).

### Haematoxylin and eosin and (HE), immunohistochemistry (IHC) and histopathological analyses

H&E staining and IHC staining were performed as described previously [19]. All immunoscores in this study were scored independently by two senior pathologists who were unaware of clinical information.

The immunostaining score for ISG15 was assessed based on both the number of positive cells as well as the intensity of staining [16]. Patients were divided into three groups (low expression group 0–3, medium expression group 4–6 and high expression group 8–12) according to the ISG15 immunoscore. The mean score was given according to the proportion of lymphocytes at high magnification 0–100%. The level of PD-L1 expression is assessed by the tumour percentage score (TPS), which represents the percentage of tumor cells with positive membrane PD-L1 staining to the total number of tumor cells at any intensity.

### Statistical analyses

The expression data of CD4^+^ and CD8^+^ T cells in ISG15 High/low cancer tissues were obtained from the Meta Data set and TCGA dataset. In this study, all experiments presented were repeated at least 3 times. All results from the statistical analysis in the text are expressed as mean ± standard deviation (mean ± SD). Chi-square test, welch’s t-test, two-tailed Student’s t test were used as appropriate. For graphical presentation and statistical analysis GraphPad Prism 8.0 was used. *P* < 0.05 is usually used to indicate statistical significance. (*P < 0.05, **P < 0.01, ***P < 0.001).

## Results

### High expression of ISG15 in lung adenocarcinoma show increased lymphocytes infiltration

**Fig. 1:**
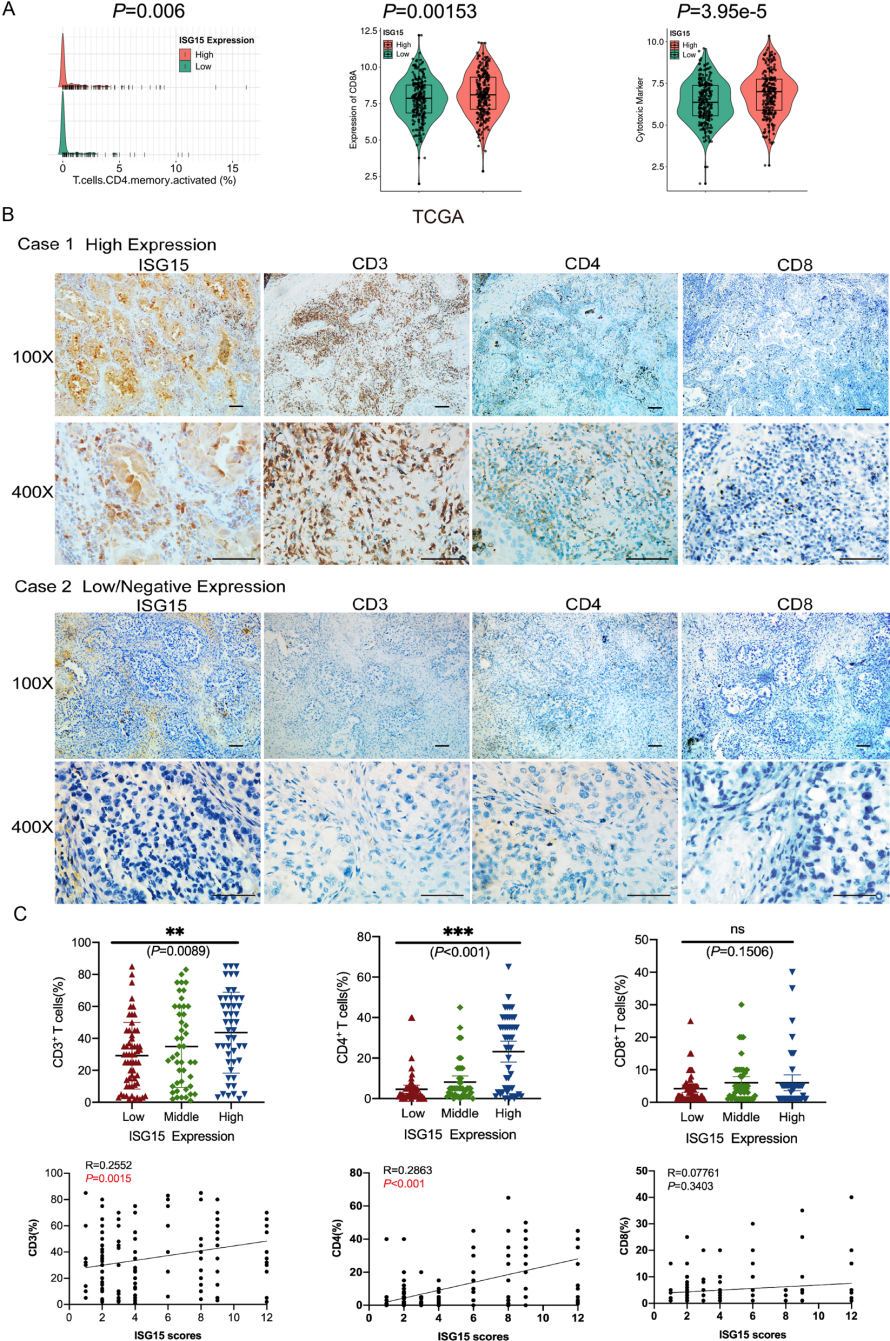
Expression of ISG15 and lymphcytes in lung adenocarcinoma. **A** Activation of memory CD4^+^ T cells, infiltration of CD8a^+^ T cells and expression of CD8^+^ T lymphocytotoxic factors in high/low ISG15 expressing tumour tissues obtained from the TCGA collection. **B** Representative IHC images showing the expression of ISG15, CD3, CD4, CD8. Scale bars 100 μm. Magnification, ×100, ×400. **C** The relationship between CD3^+^, CD4^+^, CD8^+^ T cells and ISG15 was analysed by IHC staining of LUAD

We first analyzed the TCGA dataset and found statistically significant differences in the expression of activated CD4^+^ memory T cells, CD8a^+^ in tumor cell infiltration and CD8^+^T lymphocyte cytotoxic factors in tumor tissues with high/low ISG15 expression (Fig. 1A). To further investigate the relationship between ISG15 and lymphocyte infiltration in lung adenocarcinoma, ISG15 and CD3^+^, CD4^+^, CD8^+^, CD45RA^+^, CD45RO^+^, CD20^+^, CD66b^+^, CD57^+^, Foxp3^+^, CD68^+^ microenvironmentally infiltrated lymphocytes were immunohistochemically stained in 153 prognostically intact LUAD samples (Additional file 1: Fig. S1, Additional file 2: Fig. S2, Additional file 3: Fig. S3 and Additional file 4: Fig. S4). We first analyzed the relationship between the expression of lymphocytes and the histological type of lung adenocarcinoma and found that lymphocyte infiltration in LUAD tissue did not correlate with histological type (Additional file 1: Fig. S1). We next analyzed whether ISG15 expression in LUAD cells affects microenvironmental lymphocyte infiltration. We observed a significant increase in the number of lymphocytes in tumors of strong positive ISG15 expression compared to low or negative ISG15 expression (Fig. 1B), and statistical analysis showed that higher ISG15 expression in LUAD cells was positively and statistically significant correlated with microenvironmental infiltration of CD3^+^ T and CD4^+^ T lymphocytes, but not with CD8^+^ lymphocytes or other types of lymphocytes (Fig. 1C, Fig. S3 and S4). These findings suggested a possible interaction between ISG15 and microenvironmentally infiltrating CD3^+^ T and CD4^+^ T lymphocytes.

### ISG15 regulates T cell anti-tumor immunity and inhibits lung adenocarcinoma tumor progression in vivo

**Fig. 2:**
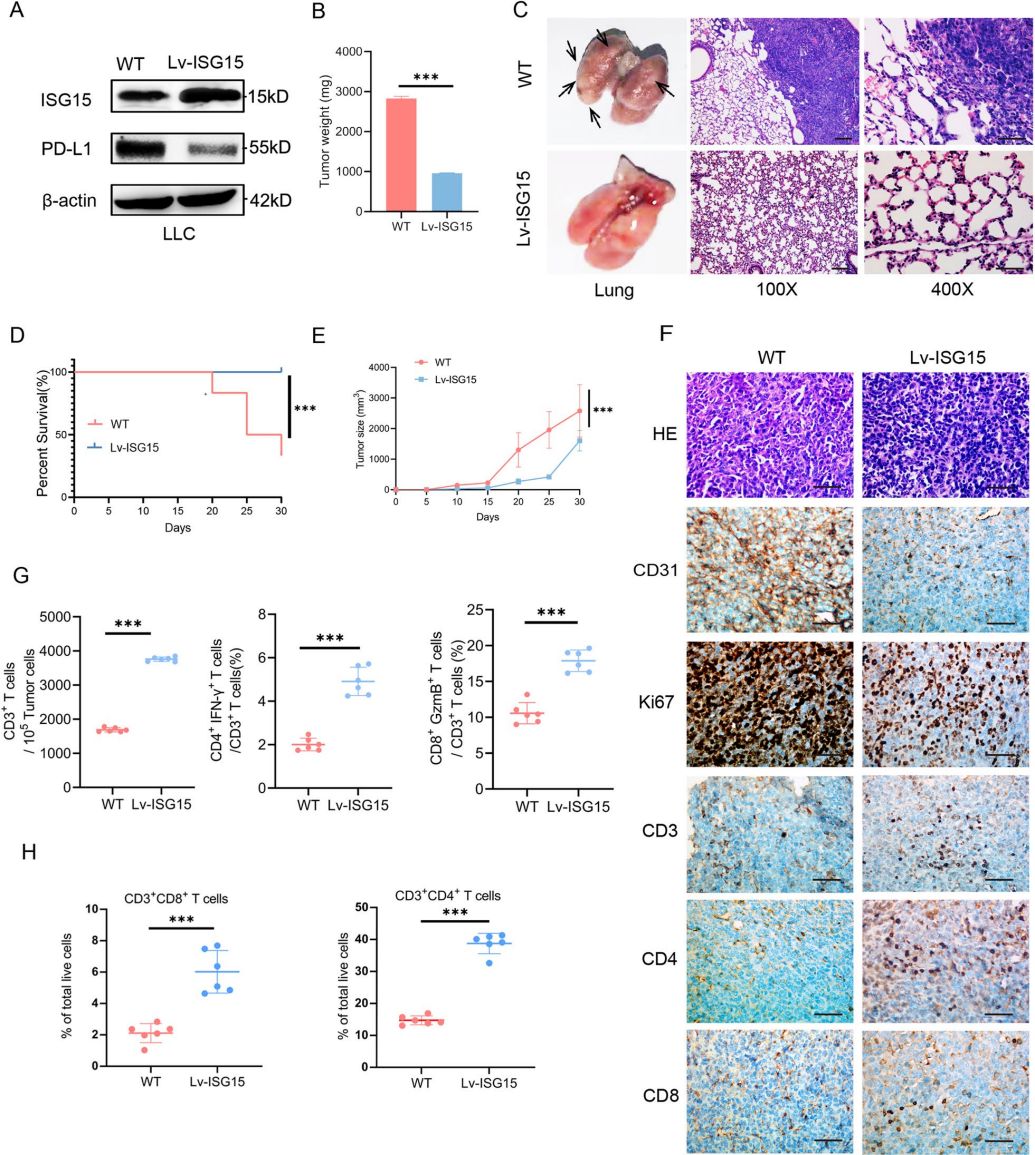
ISG15 promotes the infiltration of immune cells in vivo. **A** Western blot analysis of ISG15 in LLC with over-expressed ISG15 lentiviruses. **B** The bar graph exhibited the tumor weight of wild-type (WT) and over-expressed ISG15 (Lv-ISG15) LLC cells in immunocompetent C57BL/6 mice. **C** General views of the lungs and HE staining remove from the mice in WT and LvSG15 groups. Black arrows represent images of metastasis in the lungs. Magnification, ×100, ×400. **D** Survival curves demonstrating the WT and Lv-ISG15 groups of C57BL/6 mice during one experimental cycle (30 days). **E** The curve graph exhibited the tumor volume formation of WT and Lv-ISG15 LLC cells in C57BL/6 mice. **F** Representative images of tumors from C57BL/6 mice with HE and immunohistochemical staining of CD31, KI67, CD3, CD4, and CD8. Scale bars 100 μm. Magnification, ×400. **G** Populations of tumor-infiltrating CD3^+^ T cells and relative ratio of CD4^+^IFN-γ^+^ and CD8^+^GzmB^+^ cells in CD3^+^ TILs in WT and Lv-ISG15 groups detected by flow cytometry. **H** Populations of CD4^+^ and CD8^+^ T cells in spleen from the mice in WT and Lv-ISG15 groups detected by flow cytometry. The data are shown as the mean ± SD. *P < 0.05, **P < 0.01, ***P < 0.001

Our previous study has demonstrated the effect of ISG15 on lung adenocarcinoma in athymic nude mice [16]. To further elucidate the effect of ISG15 overexpression on lung adenocarcinoma growth through regulation of lymphocytes and in an immune-competent microenvironment, we utilized WT and stably overexpressed ISG15 (Lv-ISG15) LLC injected them subcutaneously into C57 BL/6 mice, respectively (5 mice each group, Fig. 2A). We observed that overexpressed of ISG15 significantly attenuated both the tumor size and tumor weight in contrast to the WT control cells in vivo(Fig. 2B and E) (Photographs of intact mouse subcutaneous tumors and corresponding mouse lungs are shown in Additional file 7: S7). Moreover, a shorter survival of mouse and more pulmonary metastasis of tumors were discovered in WT group (Fig. 2C and D). And this inhibition was significantly greater than the difference previously observed in nude mice [16], which indicated that ISG15 plays a critical role in activating the immune system to exert its tumor-suppressing effects.

Tumours from both groups were removed and paraffin sections were made and stained for IHC to investigate the expression of Ki-67, CD31, CD3, CD4 and CD8 in the transplanted tumours. The tumor of the WT groups, positive staining of cell proliferation marker Ki67 and angiogenesis marker CD31 were found to increase, and the number of CD3^+^, CD4^+^ and CD8^+^ tumor-infiltrating lymphocytes were significantly reduced. On the contrary, the tumor of Lv-ISG15 groups, Ki67 and CD31 staining were weak, and numbers of CD3^+^, CD4^+^ and CD8^+^ tumor-infiltrating lymphocytes were significantly reduced (Fig. 2F). For qualitative and quantitative analysis of immune cells in tumors of WT and Lv-ISG15 mice, tumor tissues were prepared as single-cell suspensions and used for flow cytometry analysis. As expected, absolute numbers of CD3^+^ TILs the relative proportion of CD4^+^IFN-γ^+^ cells among CD3^+^ TILs, and CD8^+^ granzyme B^+^(GzmB) cells were substantially increased in Lv-ISG15 tumor tissues (Fig. 2G) (The gating strategy is supplemented in Additional file 7: S7). Subsequently, to investigate whether the total number of CD3^+^CD4^+^ and CD3^+^CD8^+^ T cells was elevated in the overexpressed ISG15 mice, splenocytes from Lv-ISG15 and WT mice were subjected to flow cytometry analysis to measure the number of CD4^+^ and CD8^+^ T cells. In contrast to only the increased number of CD3^+^ and CD4^+^ cells in clinical LUAD tissues of higher ISG15 expression (Fig. 1C), we detected a significant increase in splenic CD3^+^CD4^+^ and CD3^+^CD8^+^ T cells of Lv-ISG15 mice (Fig. 2H). Based on these findings, we speculate that ISG15 mobilize activated splenic T lymphocytes to increase the number of infiltrations in the tumor, so that these T cells have anti-tumor effects into the tumor microenvironment.

### ISG15 down-regulates the expression of glycosylated PD-L1

**Fig. 3:**
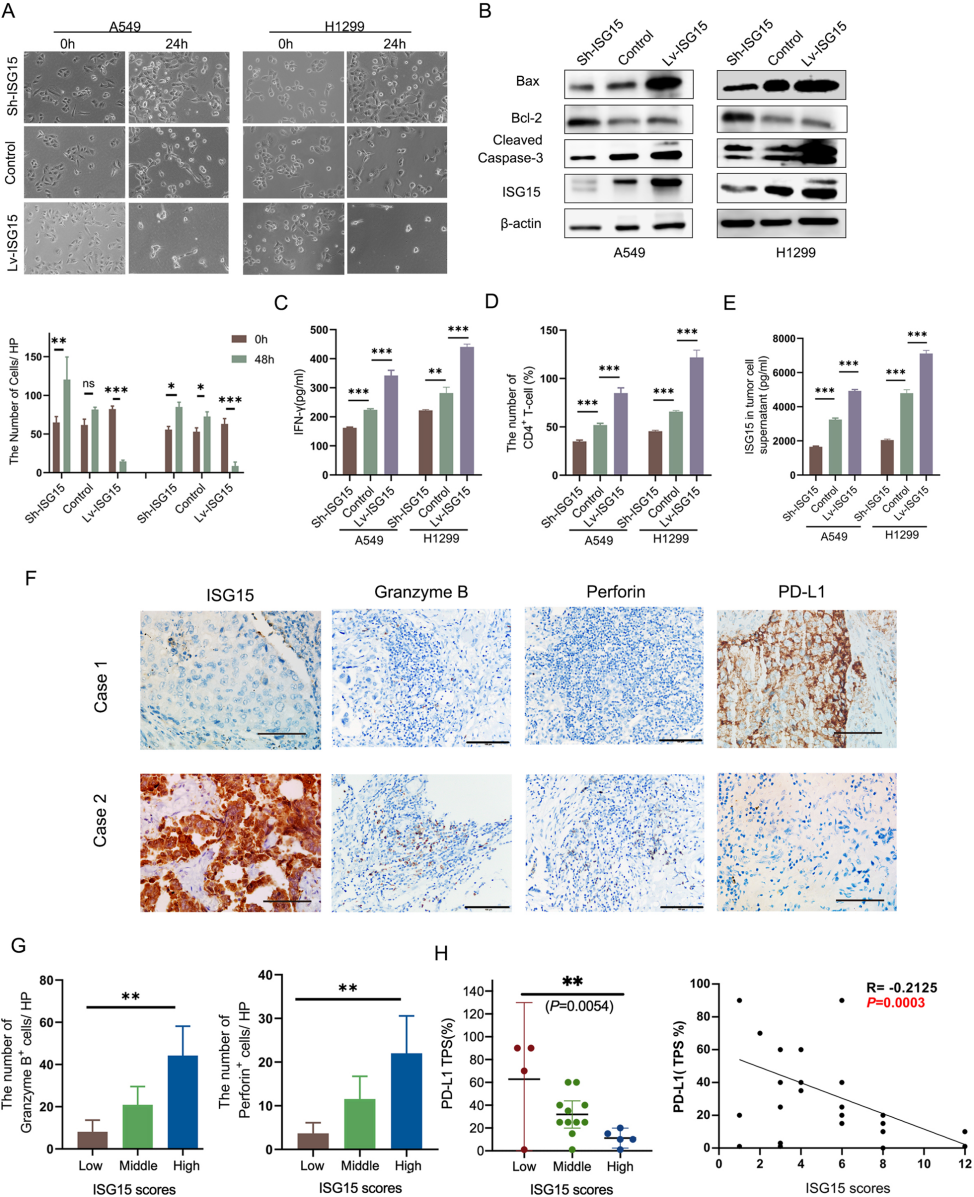
ISG15 enhances the anti-tumor immunity of CD4^+ ^T lymphocytes, which is related to PD-L1. **A** Growth of control or overexpressed with ISG15 (Lv-ISG15) or shRNA-ISG15 (Sh-ISG15) A549/ H1299 cells before and after co-culture with CD4^+^T cells. The bar chart below indicates the number of cells. **B** Western blot analysis the expression of apoptosis-related proteins in tumor cells after co-culture. **C** The IFN-γ secreted by CD4^+^ T cells co-cultured with A549 and H1299 under the three treatment conditions were evaluated by ELISA. **D** The expansion of CD4^+^ T cells co-cultured with irradiated A549/ H1299 cells or A549/ H1299 cells overexpressed/shRNA of ISG15 were assessed by CCK-8. **E** The secretion of ISG15 in the supernatant of tumor cells under three treatment conditions detected by ELISA. The data are shown as the mean ± SD. **F** Typical images of IHC staining for ISG15, granzyme B, perforin and PD-L1 in two patients with LUAD. Scale bars 100 μm. Magnification, ×400. **G** Number of cells positive for Granzyme B or Perforin in each high magnification (×400) field of view. H, correlation analysis of PD-L1 and ISG15 expression in 40 lung adenocarcinoma tissues. *P < 0.05, **P < 0.01, ***P < 0.001

Based on the conclusion that high expression of ISG15 is positively correlated with the number of CD4^+^T lymphocytes infiltrates in humans and mice, and there has been no report on the correlation between ISG15 and CD4^+^ T lymphocytes, we then focused on exploring the interaction between them. After obtaining PBMC from the peripheral blood of healthy donors, CD4^+^ T lymphocytes were isolated and cultured with 10 Gy-irradiated H1299 and A549 cells (or) overexpressed with ISG15 (Lv-ISG15) or shRNA-ISG15 (Sh-ISG15). Interestingly, we found the apoptosis of tumor cells in the Lv-ISG15 group was more obvious after co-culture than in the Sh-ISG15 group (Fig. 3A).

**Fig. 4:**
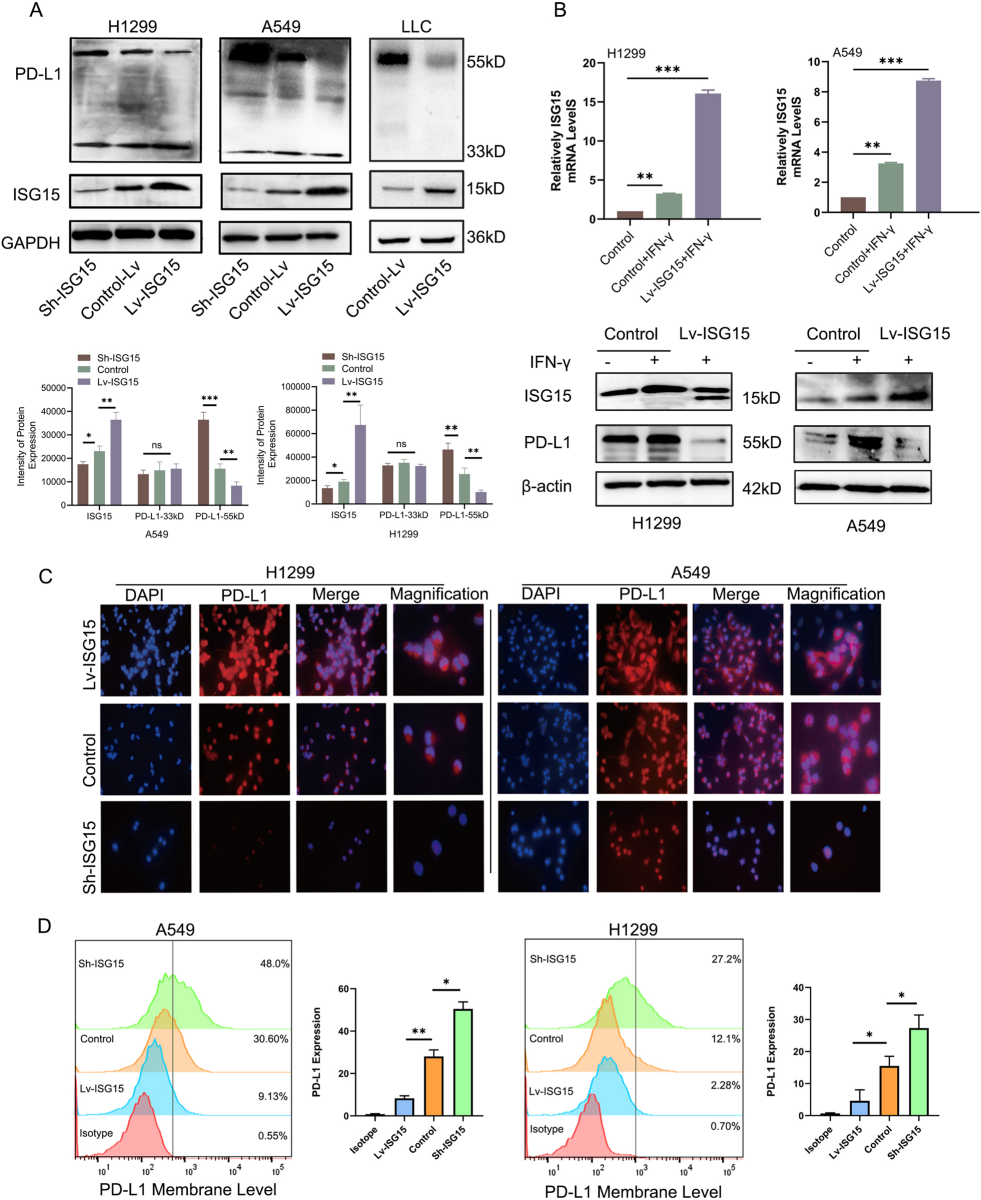
ISG15 down-regulates the expression of glycosylated PD-L1. **A** Western blot analysis of the protein level of PD-L1 in control or Lv-ISG15 or Sh-ISG15 A549, H1299 and LLC cells. **B** After 5 ng/ml IFN-γ treatment of cells in the control group or Lv-ISG15 group for 12 h, mRNA levels of ISG15 were measured by quantitative qRT-PCR and protein expression levels of PD-L1 were measured by Western blot. **C** Cellular immunofluorescence staining was used to observe the expression of PD-L1 in Sh-ISG15 and Lv-ISG15 or the control A549/H1299 cells. **D** The plasma membrane PD-L1 in control or sh-ISG15 A549/H1299 cells was detected by flow cytometry. The data are shown as the mean ± SD. Ns means no significance. *P < 0.05, **P < 0.01, ***P < 0.001

We also harvested a small fraction of co-cultured tumor cells for Western blot and found that ISG15-overexpressed cells showed higher expression of active caspase-3 and Bax proteins and lower expression of Bcl-2 than ISG15-depleted cells (Fig. 3B). This suggests a trend towards apoptosis in the cells of the Lv-ISG15 group in tumour cells co-cultured with PBMC. Besides, CD4^+^ T-cell expansion and the amount of IFN-γ in co-culture cells were detected and found that increased ISG15 expression in LUAD cells induce lymphocyte proliferation, also could enhance IFN-γ secretion by Th1-CD4^+^ T lymphocytes (Fig. 3C and D), whereas these were blocked in Sh-ISG15 of H1299 and A549 cells. Considering that ISG15 can be secreted extracellularly and the amount of ISG15 exocytosis increases with its basal expression (Fig. 3E), We hypothesized that increased ISG15 secretion might contribute to the differentiation and proliferation of Th1-type CD4^+^ T lymphocytes, thus enabling them to kill more target tumor cells. To test this hypothesis, we performed IHC staining for ISG15, granzyme B and perforin in 10 randomly selected patients with LUAD. The results elucidated that the patients with high expression of ISG15 showed positive staining for Granzyme B and Perforin, indicating that the lymphocytes had stronger killing effect on tumor cells (Fig. 3F and G). The I-IFN pathway is closely related to the regulation of PD-L1 [8], and ISG15 is an important molecule in the I-IFN pathway [9], so we speculate that ISG15 is most likely related to PD-L1. We thus further explore the relationship of ISG15 and PD-L1. Firstly, PD-L1 and ISG15 protein expression in 40 LUAD specimens were assessed by IHC. Figure 3F is a representative image of PD-L1 and ISG15 low/negative and high staining. IHC staining analysis showed a negative correlation between PD-L1 protein levels and ISG15 (Fig. 3H). Western blot revealed that depletion of ISG15 caused substantial upregulation of glycosylated PD-L1, while overexpression of ISG15 significantly decreased the glycosylated PD-L1. Surprisingly and interestingly, ISG15 did not affect non-glycosylated PD-L1 expression (33 kDa) (Fig. 4A). Similar to the results in LUAD cells, the expression of PD-L1 (55 kDa) in mouse LLC cells was also decreased with the increase of ISG15. As a key molecular in the IFN pathway, ISG15 not only responds to I-IFN, but IFN-γ can also increase the level of ISG15 mRNA (Fig. 4B). As IFN-γ produced by activated T cells can upregulate PD-L1 on the surface of tumour cells [20], we were interested in whether ISG15 could influence induced PD-L1 expression. As a result, upregulation of ISG15 significantly attenuated the IFN-γ-induced glycosylated PD-L1 expression than control in H1299 and A549 cells (Fig. 4B). This result means that the inhibition of PD-L1 by ISG15 reversed the promotion of PD-L1 by IFN-γ. The fluorescence intensity of PD-L1 was clearly decreased in the LV-ISG15 group by immunofluorescence (Fig. 4C). As shown by flow cytometry, there was a substantial increase in PD-L1 expression on the cell surface of the Sh-ISG15 group compared to Isotype, while Lv-ISG15 expression was significantly decreased (Fig. 4D).

**Fig. 5:**
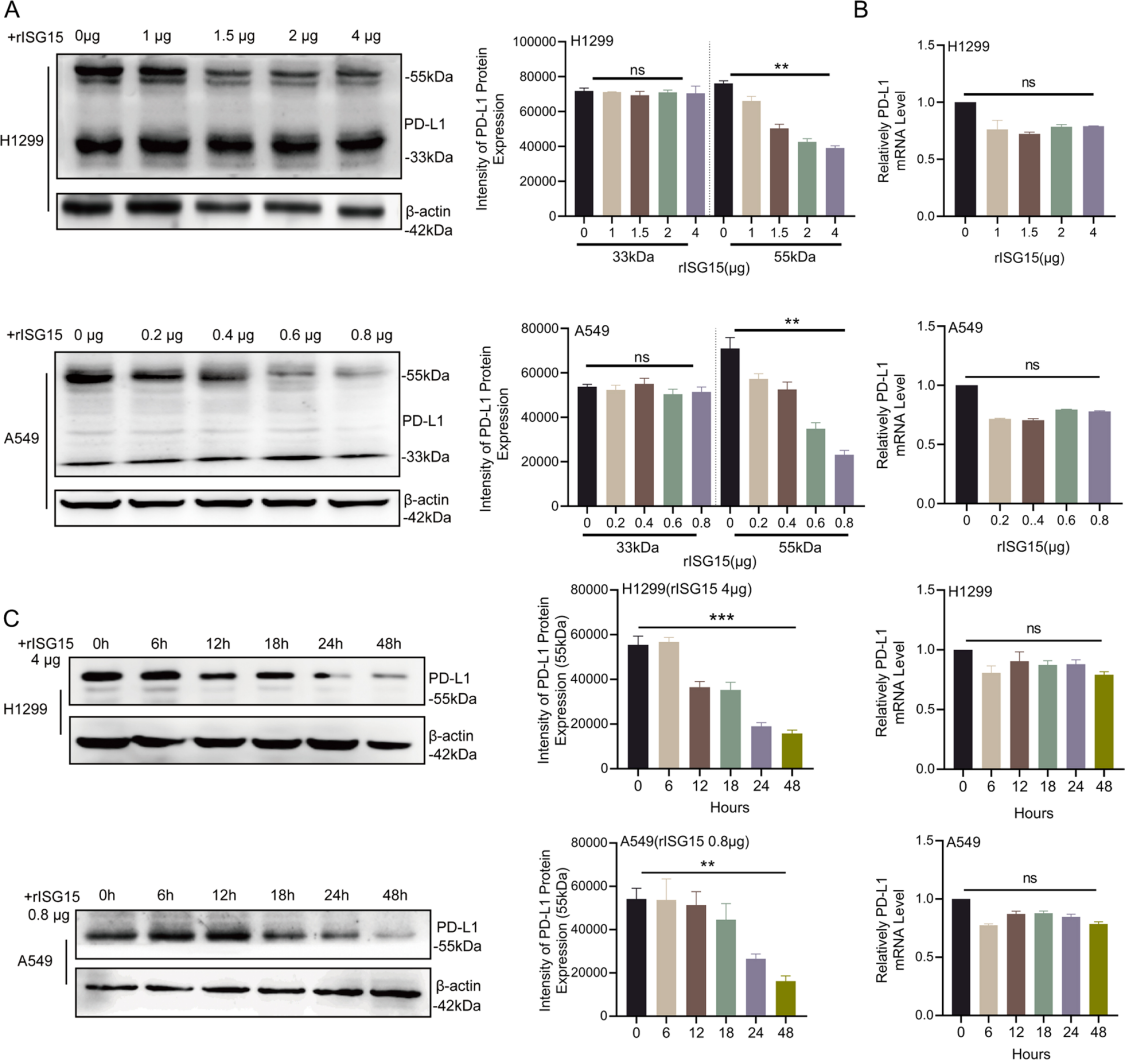
Exogenous ISG15 also reduces the expression of glycosylated PD-L1. **A** Western blot and RT-qPCR were used to detect the expression of different molecular weights of PD-L1 at concentrations of 0 μg, 1 μg, 1.5 μg, 2 μg, and 4 μg of exogenous ISG15 in A549 and H1299. **B** H1299 or A549 treated with 4 μg or 0.8 μg of exogenous ISG15, the changes of PD-L1 with different molecular weights (left panel) and mRNA level (right panel) in 0–48 h.The data are shown as the mean ± SD. Ns means no significance. *P < 0.05, **P < 0.01, ***P < 0.001

In addition, we also treated H1299 and A549 cells with exogenous ISG15. Western blot revealed that exogenous ISG15 could similarly attenuate glycosylated PD-L1 and with little effect on non-glycosylated PD-L1 in a dose-dependent manner (Fig. 5A). The expression of glycosylated PD-L1 was also decreased when the cells were treated in a time-dependent manner at the highest concentration of exogenous ISG15 (Fig. 5C). As expected, in line with the prior results, exogenous ISG15 also did not work on PD-L1 mRNA (Fig. 5B and D). These results suggest that exogenous ISG15 may be incorporated into tumour cells and play a similar role to endogenous ISG15 in reducing glycosylated PD-L1.

### ISGylation targets PD-L1 promote its ubiquitination for proteasome-dependent degradation

It was unclear from the results that whether the reduced glycosylated PD-L1 is due to ISG15 reducing its formation or promoting its degradation. Given the paucity of reports related to the regulation of ISG15 and glycosyltransferases, and that ISG15 can act as a ubiquitination-like molecule to regulate target proteins [12], we will focus on the speculation of the degradation effect of ISG15 on PD-L1 in the following research.

**Fig. 6:**
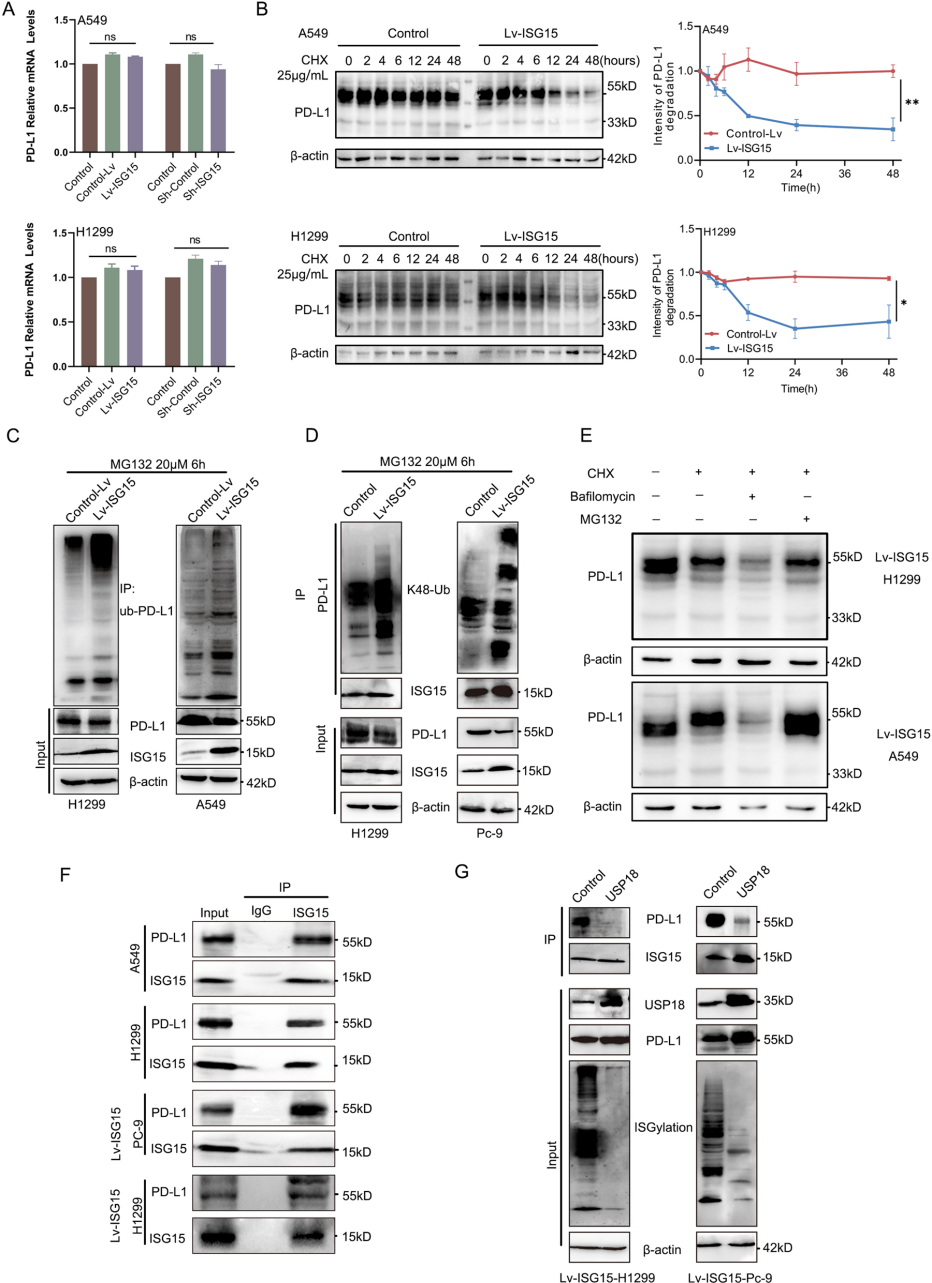
ISG15 and PD-L1 form ISGylation and promote its ubiquitination for proteasome-dependent degradation. **A** Quantitative qRT-PCR analysis of the mRNA level of PD-L1 in Sh-ISG15 and Lv-ISG15 or the control A549/H1299 cells. **B** Western blotting was performed to detect the expression level of glycosylated PD-L1 at different times in A549 and H1299, after CHX (25 μg/ml) treatment. The data were normalized for comparative purposes (bottom panel). **C** Ubiquitination level of PD-L1 detected by Immunoprecipitation (IP) assay in Lv-ISG15 or control cells after treated with MG132 (20 μmol) for 8 h. **D** Immunoprecipitation of ISG15 and PD-L1 at lysine48-linked ubiquitination sites after H1299 and Pc-9 treated with 20 μM MG132 6 h. **E** Western blot was used to examine the effect of bafilomycin or MG132 on PD-L1 levels in the presence of CHX in Lv-ISG15 cells. **F** IP experiment detects the interaction between ISG15 and PD-L1. **G** Co-IP assay detects whether the interaction between ISG15 and PD-L1 is specific to ISGylation and whether it is dissociated by USP18 in H1299 and Pc-9 cells co-transfected ISG15 and USP18 or control plasmids

Initially the mRNA expression of PD-L1 at different ISG15 expression levels was assessed. The mRNA levels of PD-L1 were not significantly related to the ISG15 protein expression profile according to RT-qPCR results. To further elucidate the mechanism of ISG15-mediated PD-L1 downregulation, we exposed A549 and H1299 to the protein translation inhibitor cycloheximide (CHX). Consistent with our expectation, glycosylated PD-L1 was degraded more rapidly in Lv-ISG15 cells than in control cells in the presence of CHX (Fig. 6B), suggesting that ISG15-induced PD-L1 downregulation is predominantly controlled at the protein level. As the degradation of PD-L1 is closely related to its ubiquitination, we examined the ubiquitination of PD-L1 in the presence of CHX. As ISG15 increased, the level of PD-L1 ubiquitination also increased (Fig. 6C), and its K48-linked chain was at high expression levels in cells overexpressing ISG15 (Fig. 6D). Since the K48-linked ubiquitin chain can act as a proteasomal degradation signal, this seems to imply that PD-L1 may be degraded via the proteasomal pathway. LUAD cells were treated with either the proteasome inhibitor MG132 or the lysosomal inhibitor bafilomycin to determine the degradation pattern of glycosylated PD-L1. We found that the degradation of PD-L1 was restored by MG132 (Fig. 6E). Therefore, it can be inferred that ISG15 promotes the ubiquitination of PD-L1 and thus its degradation by the ubiquitin–proteasome pathway. Next, we wanted to further explore whether the increase in ISG15-induced PD-L1 ubiquitination was caused by an interaction between them. Immunoprecipitation (IP) showed that PD-L1 could be detected when the ISG15 antibody was pulled down (Fig. 6F). So, is this interaction specific to ISGylation and is PD-L1 the target protein of ISG15? We chose another PC-9 cell with high ISG15 expression, which had a more stable effect.

Both H1299 cells, which overexpress ISG15, and PC-9 cells, transfected with the USP18 plasmid, which is specific for the dissociation of ISGylation, were selected. Interestingly, as we expected, the results of the Co-IP experiments showed that the interaction between PD-L1 and ISG15 gradually diminished or even disappeared as the dissociation of ISGylation gradually increased (Fig. 6E). Taken together, these data identify that it is precisely is the formation of K-48-modified ISGylation between ISG15 and glycosylated PD-L1 that leads to increased ubiquitination of PD-L1 and consequently increased degradation of the ubiquitin–proteasome pathway targeting glycosylated PD-L1.

### ISG15 elevation-induced destabilization of PD-L1 improve PD-L1-targeted immunotherapy

**Fig. 7:**
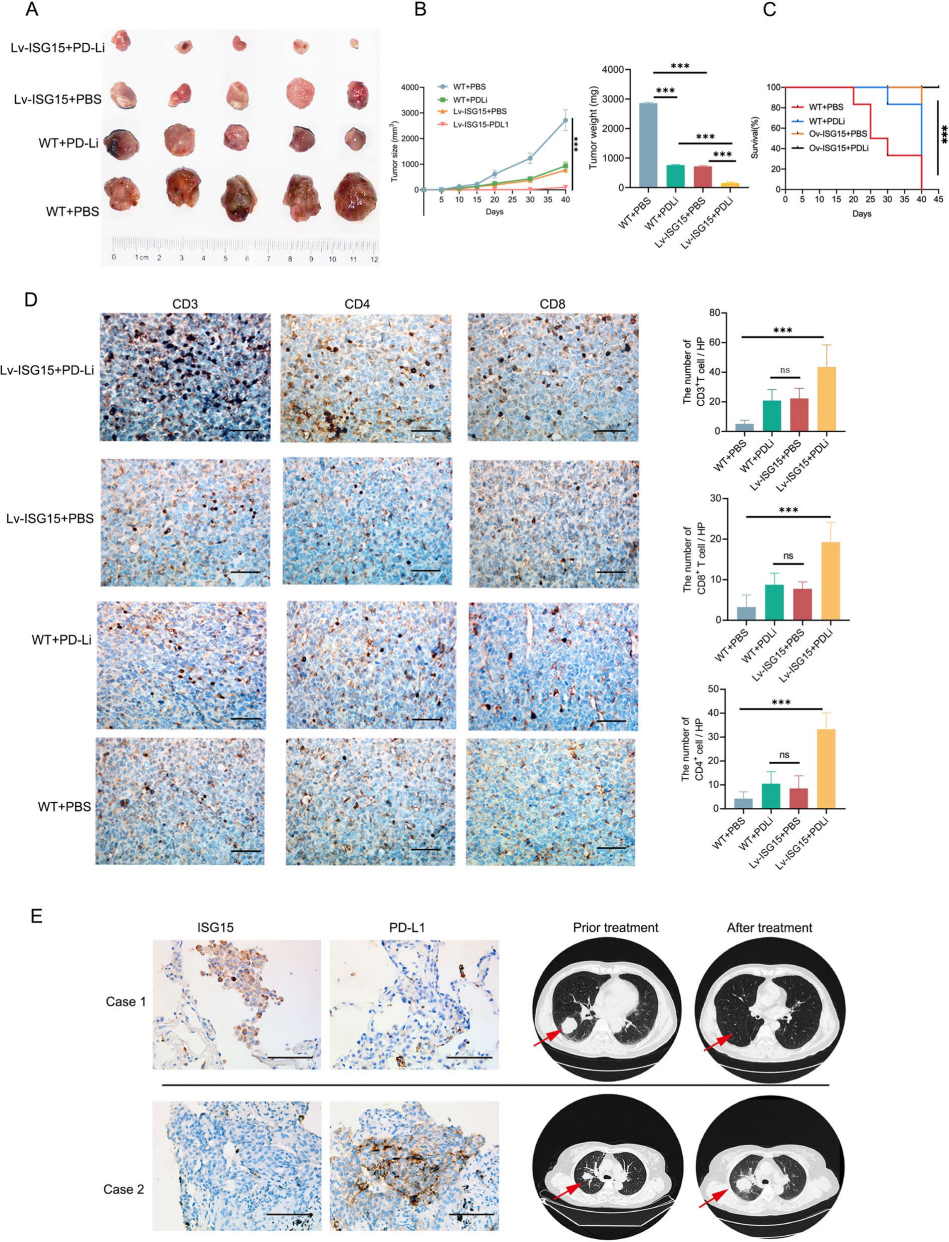
ISG15 elevation-induced destabilization of PD-L1 improve PD-L1-targeted immunotherapy

Since PD-L1 undergoes ISGylation of ISG15, induction of PD-L1 destabilization may improve PD-L1-targeted immunotherapy. To explore the impact of ISG15 on immune checkpoint blockade responses, we finally treated WT and Lv-ISG15 C57BL/6 mouses with PBS and anti-PD-L1 antibody (PDLi). The results showed that the growth and weight of immunotherapy WT tumors were similar to Lv-ISG15 tumors (Fig. 7A and B). Interestingly, anti-PD-L1 antibody showed outstanding effects in tumors formed by LLC cells overexpressing ISG15, indicating that ISG15 elevation facilitates antitumor immune response. In addition, we also observed that the combination of ISG15 overexpression and anti-PD-L1 antibody significantly prolonged the survival of mice compared to other single treatment or PBS control groups (Fig. 7C). Subsequently, we performed IHC staining of CD3^+^, CD4^+^ and CD8^+^ T lymphocytes on these four groups of tumors. The results showed that the tumors in the Lv-ISG15 + PDLi group showed a large amount of CD3^+^, CD4^+^ and CD8^+^ T lymphocyte infiltration, while the difference between the WT + PDLi group and the Lv-ISG15 + PBS group was not statistically significant. Based on these data, we speculate that ISG15 recruit T lymphocytes on one hand, and on the other hand, since ISG15 can decrease PD-L1 in tumor cells, thus allowing these T cells with antitumor effects to enter the tumor microenvironment without inhibiting their activity and exerting antitumor immunity.

Subsequently, we collected 18 NCLSL patients treated with anti-PD-L1 antibody for PD-L1 and ISG15 staining analysis, and the results were shown in Additional file 5: S5. The analysis revealed that although some patients had low or negative PD-L1 expression in their tumour tissue, anti-PD-L1 antibody therapy was still effective in achieving complete clinical remission due to their high ISG15 expression. Patients who progressed despite anti-PD-L1 antibody were all negative for ISG15 (Fig. 7E). Therefore, while not everyone who is effectively treated with anti-PD-L1 antibody is ISG15-positive, ISG15-positive anti-PD-L1 antibody-treated patients may have better outcomes.

## Discussion

ISG15 was the first ubiquitin-like protein (Ubl) to be identified and acts not only in the unconjugated/conjugated form inside the cell, but also in the free form outside the cell [10, 21]. ISG15 and the enzymes that catalyze ISGylation and de-ISGylation are dysregulated in many types of cancer, including breast, pancreatic, and prostate cancers [14, 22–24]. However, it remains uncertain whether ISG15 and ISGylation play a pro-tumor or anti-tumor role in cancer. Our recent studies suggest that high ISG15 expression usually predicts a better prognosis in LUAD. Mechanistically, ISG15 forms ISGylation with ESRP1 and stabilizes ESRP1, co-repressing the EMT [16]. In this study, we demonstrated that ISG15 is also responsible for post-translational modification of PD-L1. ISG15 and PD-L1 form ISGylation which involving k-48 ubiquitin-chain and then destabilizes glycosylated PD-L1 and promotes its degradation through the ubiquitin–proteasome pathway, thus activating anti-tumor immune function. Whether ISG15 stabilizes target proteins or degrades them may depend on the different ubiquitin-chain modifications during ISGylation formation of ISG15 with different target proteins. The underlying mechanism for this difference may merit future in-depth study.

Previous studies have demonstrated that extracellular ISG15 not only acts as an immune adjuvant to enhance the anti-tumour immunity of CD8^+^ T cells, but also activates NK cells and increases their production of IL-2 and IFN-γ cytokines, resulting in the proliferation of ovarian cancer CD8^+^ T cells. Our data from tumor cell and CD4^+^ T lymphocytes co-culture experiments showed that ISG15 can stimulate the differentiation of Th1 subtype CD4^+^ T cells and a elevated secretion of IFN-γ. These data are similar to another report that USP18-deficient mammary epithelial cells induce recruitment of Th1 subtype CD4^+^ T cells by up-regulating IFN-γ secretion of Cxcl10, forming a tumor-inhibiting microenvironment [25].

And we also confirmed that over-expression of ISG15 can increased the number of CD4^+^ and CD8^+^ T cells in spleen and the cytotoxicity of tumor-infiltrating lymphocytes in Lewis’s xenograft models. In this study, we present a new result showing that glycosylated PD-L1 was also down-regulated by exogenous ISG15 in a dose- and time-dependent manner in human LUAD cells. Although it is interesting to note that the two cell lines, A549 and H1299, differ in their sensitivity to exogenous ISG15. What remains unclear is whether the reduction of glycosylated PD-L1 by exogenous ISG15 is due to integration into cells that also exert ISGylation effects or some other possible mechanism. Perhaps, exogenous ISG15 enters the cell as free intracellular ISG15 and binds to the target protein in a non-covalent manner to regulate its activity, as previously reported [26, 27]. In any case, ISG15 may be considered in the future as a small molecule immunoadjuvant to better inhibit cancer progression in patients who are not susceptible to immunosuppressive therapy.

Glycosylation is essential for PD-L1, on the one hand glycosylated PD-L1 is more stable and on the other hand glycosylated PD-L1 regulates PD-L1/PD-1 interactions and mediates immunosuppression [17, 18]. We here first discovered that ISG15 can decrease glycosylated PD-L1 but not non-glycosylated PD-L1. Additionally, depletion or elevation of ISG15 does not affect the accumulation or depression of PD-L1 mRNA. We also clarified for the first time that Ubiquitination modification of PD-L1 by ISG15 is due to the formation of specific ISGlation, which can be blocked by USP18. Our study focuses on the degradation of glycosylated PD-L1 by ISG15, and it is possible that ISG15 has a common role in reducing the formation of glycosylated PD-L1. It may be that the ubiquitination modification of PD-L1 by ISG15 affects the glycosylation modification of PD-L1 in its spatial location, creating a competitive inhibition of it and thus leading to a decrease in glycosylated PD-L1 level. Our future research may shed light on these interesting speculations.

There are still some shortcomings in this paper, one of which is that it is difficult to confirm the relationship between ISG15 and glycosylated PD-L1 in tissue specimens. It has been reported that structural hindrance of N-glycan to PD-L1 in fixed samples prevents its recognition by PD-L1 diagnostic antibodies [28–30], making it difficult for us to accurately observe the effect of ISG15 on glycosylated PD-L1 in tissues. This may also be the reason why in Additional file 5: S5, although some patients have low ISG15 expression, their PD-L1 expression is not high. It is therefore crucial to stratify patients according to ISG15 expression in order to determine which patients are likely to benefit most from PD-L1 inhibitors and thus optimise efficacy.

In a nutshell, our study shows ISG15 is a newly discovered post-translational modification molecule of PD-L1. PD-L1 instability induced by elevated ISG15 improved PD-L1-targeted immunotherapy and inhibited LLC tumor growth in vivo, suggesting a possible strategy to target PD-L1-mediated immune escape of tumor cells. These findings identify ISG15 as a key node in the regulation of PD-L1 stability and that ISG15 has a strong potential to enhance tumour-specific immunity.

## Supplementary Information

**Additional file 1: Fig. S1.** The relationship between pathological types of lung adenocarcinoma and T cell lymphocytes. A–F, The relationship between the expression of CD3^+^, CD4^+^, CD8^+^, CD45RO^+^, CD45RA^+^ and Foxp3^+^ T lymphocytes in five pathological types of lung adenocarcinoma.

**Additional file 2: Fig. S2.** The relationship between the pathological type of lung adenocarcinoma and lymphocytes other than T cells. A–D, The infiltration of CD20^+^ B lymphocytes, CD66b^+^ neutrophils, CD68^+^ monocytes and CD57^+^NK cells in lung adenocarcinoma and its relationship with five pathological types of lung adenocarcinoma.

**Additional file 3: Fig. S3.** Typical images of ISG15 and lymphocyte IHC staining. A, B, Typical IHC images of ISG15 and lymphocytes other than CD3^+^and CD4^+^ T cells.

**Additional file 4: Fig. S4.** Correlation of ISG15 with lymphocytes. A–G, The correlation between ISG15 and the expression of lymphocytes except CD3 + and CD4^+^ T lymphocytes.

**Additional file 5.** Clinical cases treated with anti-PD-L1 mab.

**Additional file 6.** Antibodies and reagents.

**Additional file 7.** Images of tumours and lungs from WT and Lv-ISG15 groups of mice and gating strategies for flow cytometry. A and B, photographs of subcutaneous tumors and corresponding mouse lungs in WT and Lv-ISG15 mice. C, gating strategies for flow cytometry of tumor tissues from WT and Lv-ISG15 mice.

## Data Availability

The datasets used or analysed during the current study are available from the corresponding author on reasonable request.

## Abbreviations

ICIs: Immunocheckpoint inhibitors
NSCLC: Non-small-cell lung cancer
LUAD: Lung adenocarcinoma
PD-L1: Programmed cell death-ligand 1
PD-1: Programmed cell death-1
IFN-I: Type I interferon
ISG15: Interferon-stimulated gene 15
ESRP1: Epithelial Splicing Regulatory Protein 1
LLC: Lewis mouse lung cancer cell line
HE: Haematoxylin and eosin
IHC: Immunohistochemistry
CHX: Cycloheximide
IP: Immunoprecipitation
PTMs: Post-translational modifications
qRT-PCR: Real-Time Quantitative Reverse Transcription PCR
Baf: Bafilomycin
