# Supplementary figures and images for: ISG15 targets glycosylated PD-L1 and promotes its degradation to enhance antitumor immune effects in lung adenocarcinoma
Source: J Transl Med. 2023 May 22;21:341. doi: 10.1186/s12967-023-04135-1 (PMC10204161; doi:10.1186/s12967-023-04135-1)

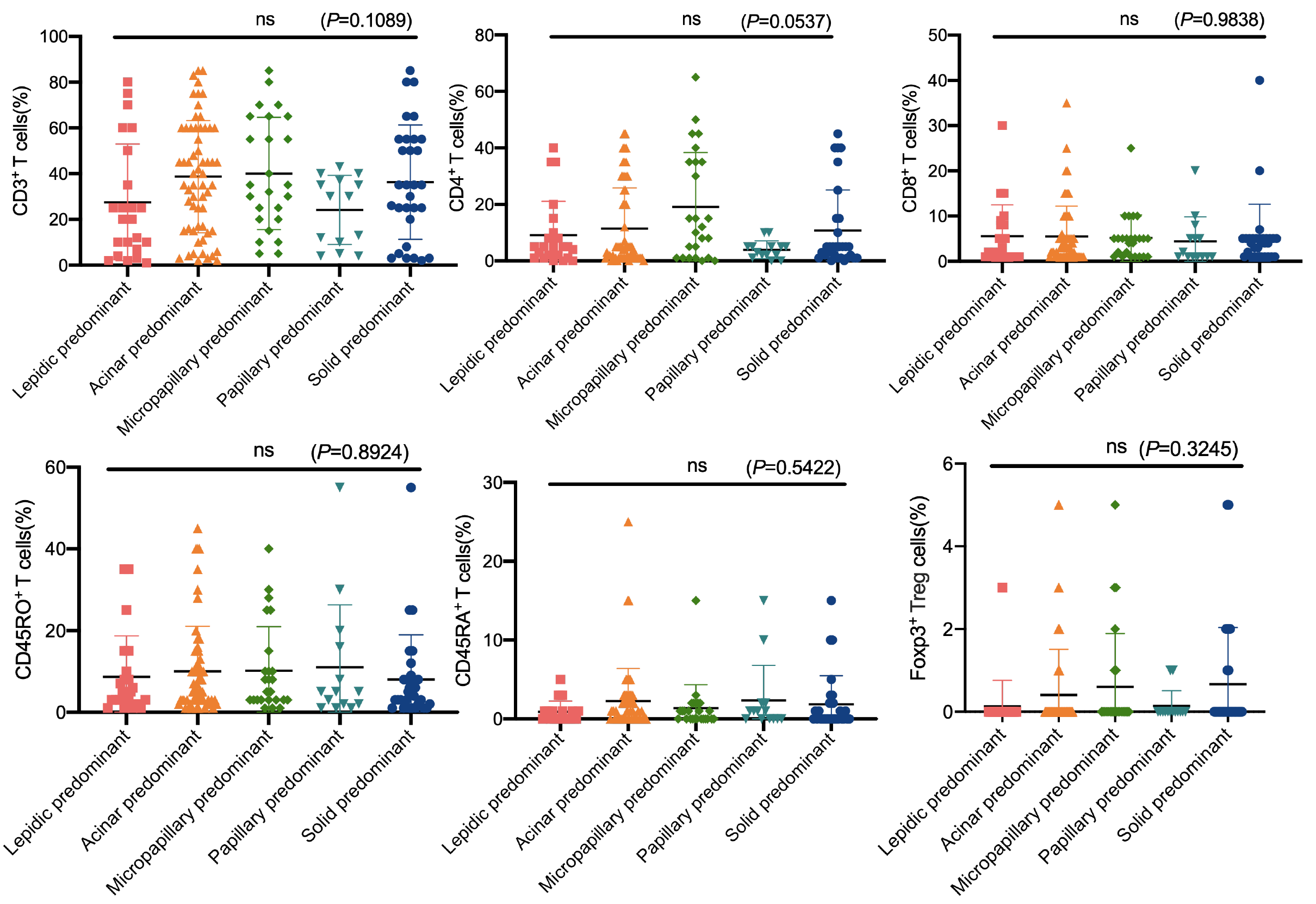

Supplement: Supplementary file 1 — Additional file 1: Fig. S1. The relationship between pathological types of lung adenocarcinoma and T cell lymphocytes. A–F, The relationship between the expression of CD3+, CD4+, CD8+, CD45RO+, CD45RA+ and Foxp3+ T lymphocytes in five pathological types of lung adenocarcinoma. [file 12967_2023_4135_MOESM1_ESM.png]

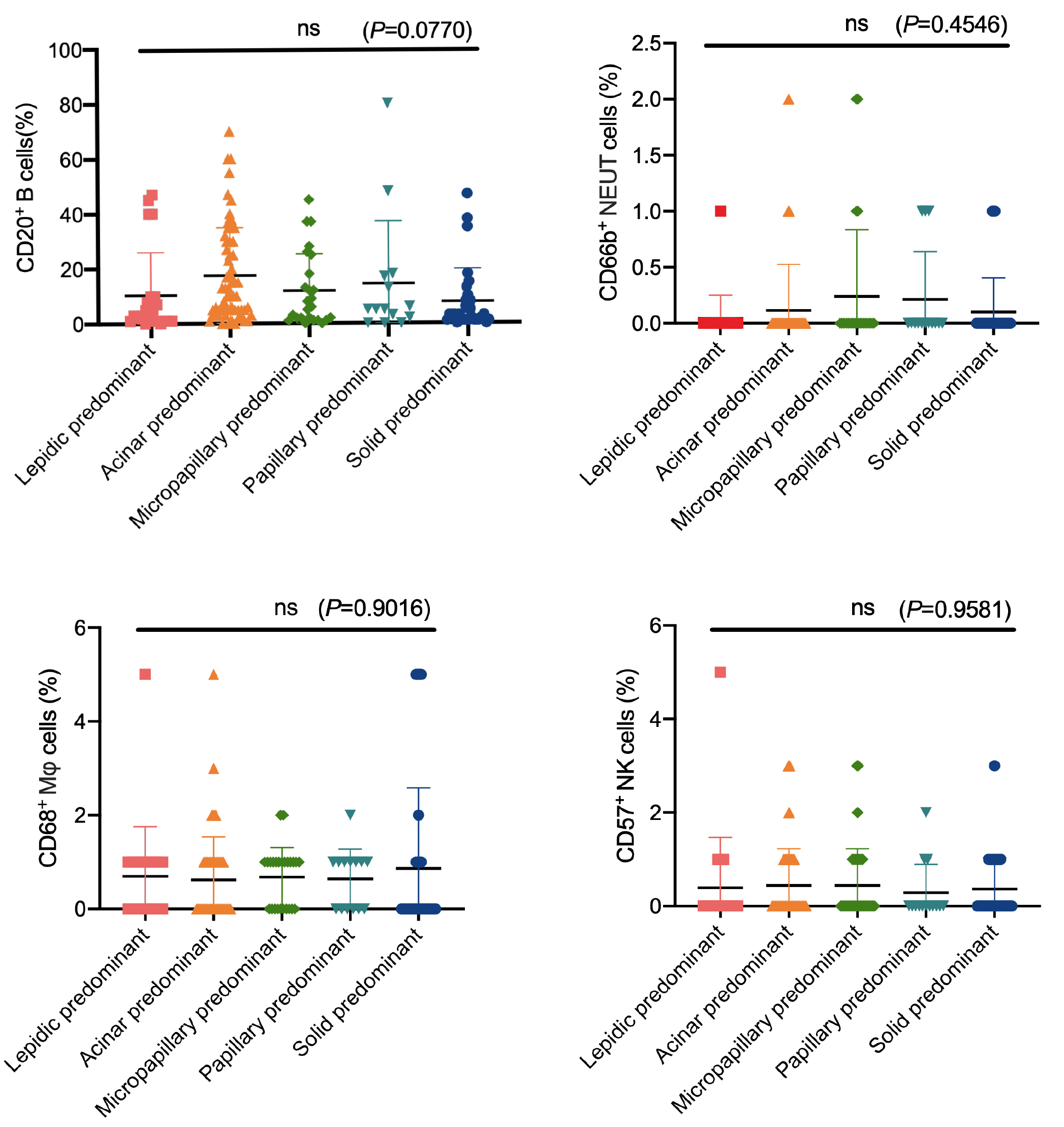

Supplement: Supplementary file 2 — Additional file 2: Fig. S2. The relationship between the pathological type of lung adenocarcinoma and lymphocytes other than T cells. A–D, The infiltration of CD20+ B lymphocytes, CD66b+ neutrophils, CD68+ monocytes and CD57+NK cells in lung adenocarcinoma and its relationship with five pathological types of lung adenocarcinoma. [file 12967_2023_4135_MOESM2_ESM.png]

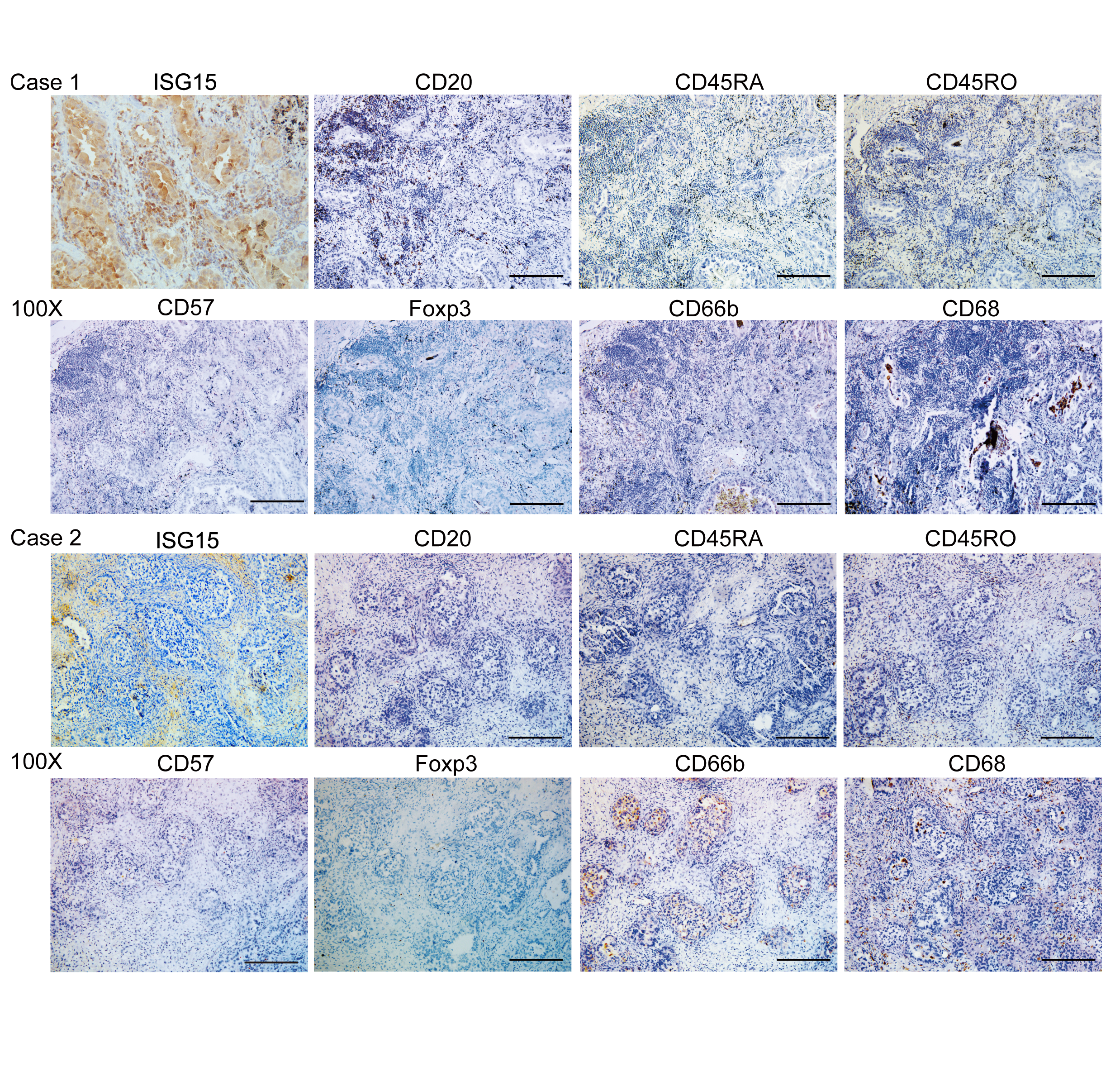

Supplement: Supplementary file 3 — Additional file 3: Fig. S3. Typical images of ISG15 and lymphocyte IHC staining. A, B, Typical IHC images of ISG15 and lymphocytes other than CD3+and CD4+ T cells. [file 12967_2023_4135_MOESM3_ESM.png]

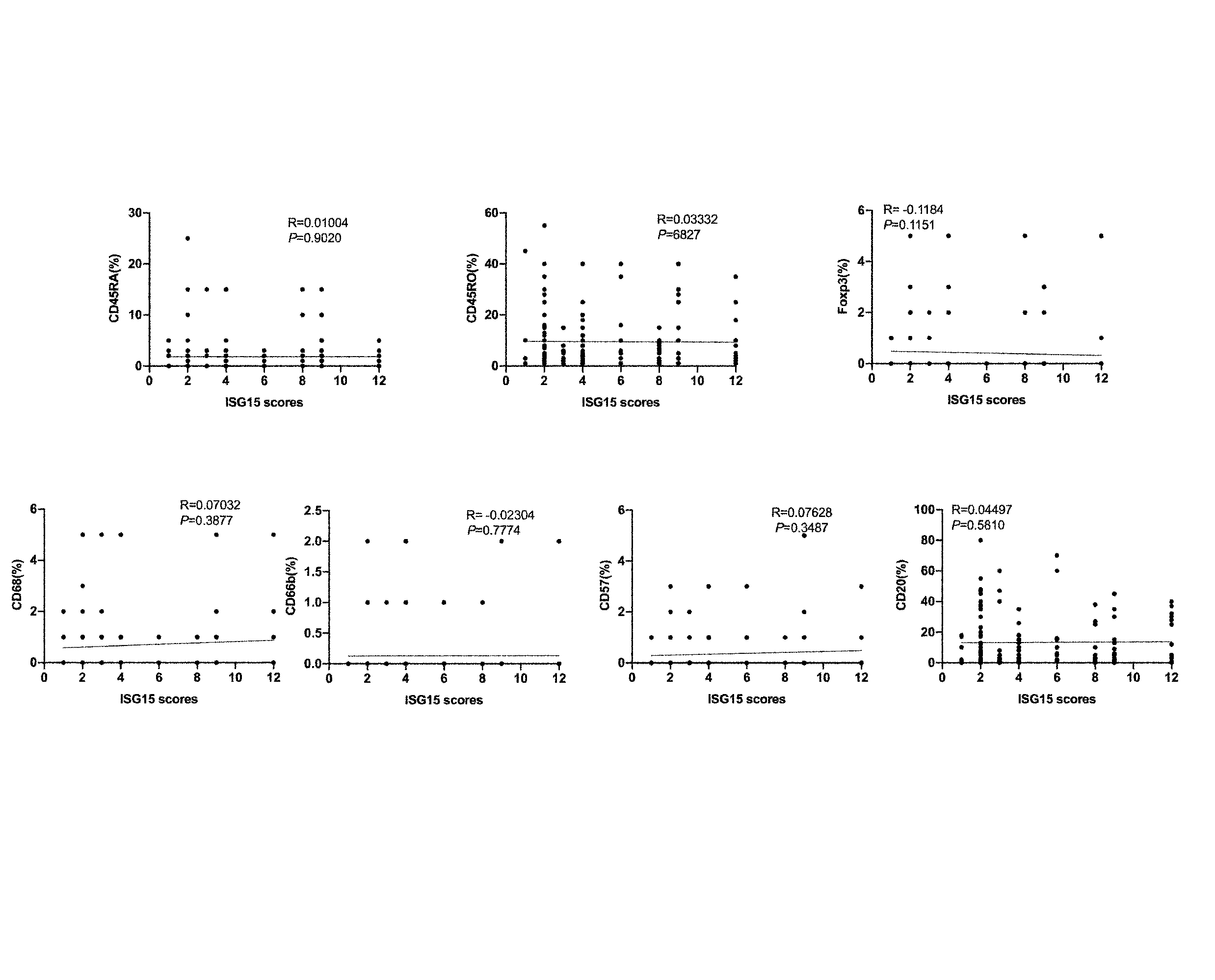

Supplement: Supplementary file 4 — Additional file 4: Fig. S4. Correlation of ISG15 with lymphocytes. A–G, The correlation between ISG15 and the expression of lymphocytes except CD3 + and CD4+ T lymphocytes. [file 12967_2023_4135_MOESM4_ESM.png]

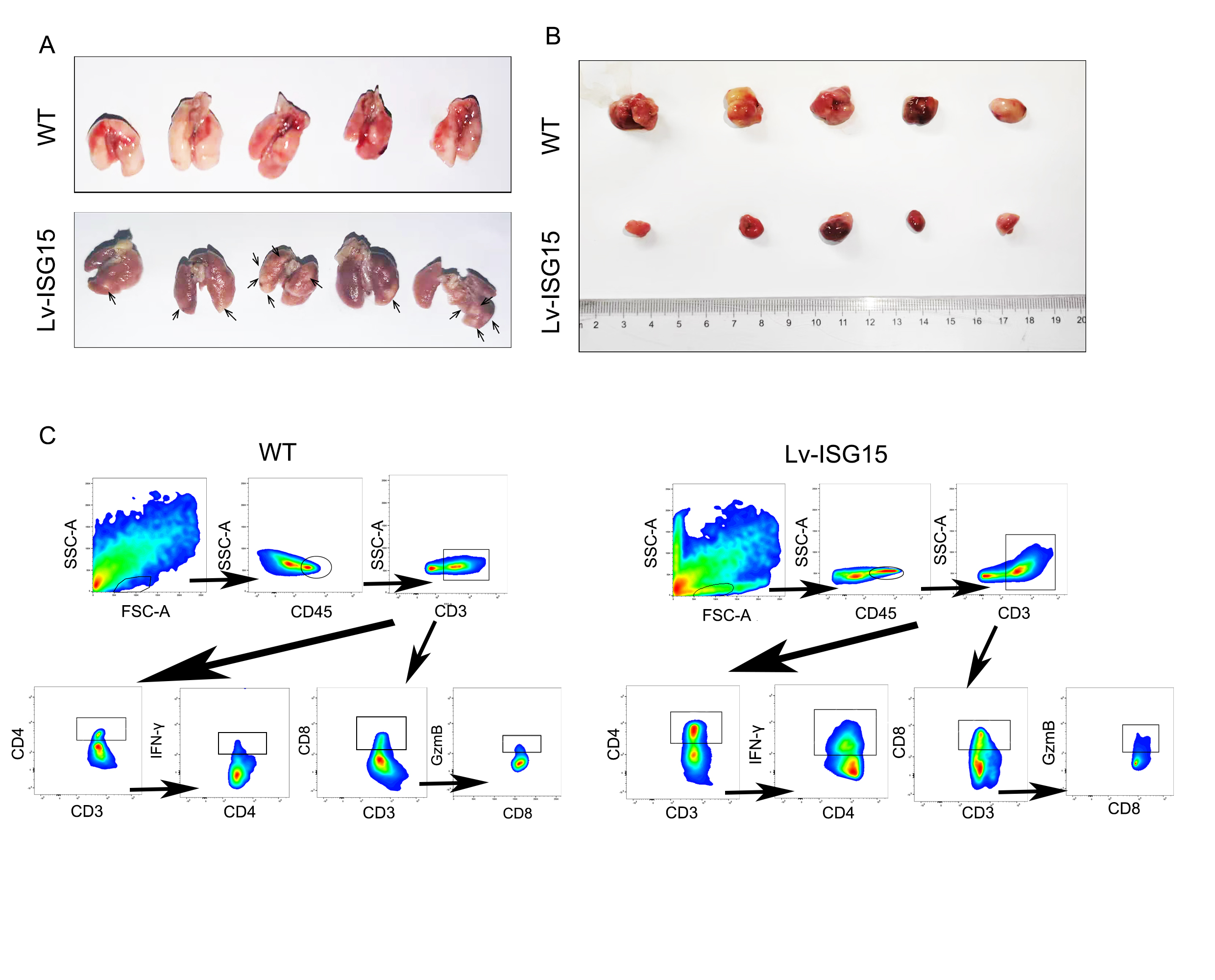

Supplement: Supplementary file 7 — Additional file 7. Images of tumours and lungs from WT and Lv-ISG15 groups of mice and gating strategies for flow cytometry. A and B, photographs of subcutaneous tumors and corresponding mouse lungs in WT and Lv-ISG15 mice. C, gating strategies for flow cytometry of tumor tissues from WT and Lv-ISG15 mice. [file 12967_2023_4135_MOESM7_ESM.jpg]
