# Supplementary material for: ISG15 targets glycosylated PD-L1 and promotes its degradation to enhance antitumor immune effects in lung adenocarcinoma
Source: J Transl Med. 2023 May 22;21:341. doi: 10.1186/s12967-023-04135-1 (PMC10204161; doi:10.1186/s12967-023-04135-1)
Supplement: Supplementary file 6 — Additional file 6. Antibodies and reagents. [file 12967_2023_4135_MOESM6_ESM.docx]

**Antibodies and reagents**

anti-ISG15 (Santa Cruz Biotechnology, sc-166755, 1:200),anti-CD3 (ZSJQ Bio, ZM-0417)，anti-CD4 (ZSJQ Bio, ZM-0418), anti-CD8 (ZSJQ Bio, ZA-0508), anti-CD20 (ZSJQ Bio,TA800385 ), anti-CD45RA (Abcam, ab755, 1:100) , anti-CD45RO (Abcam, ab23, 1:100) , anti-CD57(Cell Signaling Technology, #72031, 1:400), anti-CD66b (Abcam, ab197678, 1:100), anti-CD68(Abcam, ab955, 1:3000), anti-Foxp3 (Cell Signaling Technology, #12653, 1:400) , anti-Bax (Abcam, ab32503, 1:1000), anti-Bcl-2 (Abcam, ab32124, 1:1000), anti-cleaved-caspase 3 (Abcam, ab2302, 1:500), anti-PD-L1 (Cell Signaling Technology, #13684s, 1:1500, for WB), anti-Ub (Cell Signaling Technology, #58395s, 1:1000), anti-k48-Ub (Cell Signaling Technology, #12805s, 1:1000), anti-USP18 (Abcam, ab168478, 1:1000) , anti-β-actin (Cell Signaling Technology, #3700s,1:5000),anti-Ki-67 (Cell Signaling Technology, #12202, 1:100) , anti-CD31 (Cell Signaling Technology, #77699, 1:100), anti-CD3 (eBioscience, 11-0031-82,FITC conjugated), anti-CD4 (eBioscience, 12-0041-82, PE conjugated), anti-CD8 (Invitrogen, MCD0831, PerCP conjugated), anti-IFN-γ (Invitrogen, PM9001, FITC conjugated), anti-GzmB (eBioscience, 17-8898-82, APC conjugated), anti-PD-L1(Biolegend, 124305, APC conjugated ) , anti-Rat IgG2b (Biolegend, 400603, κ Isotype Ctrl ), anti-PD-L1 (Dako, 28-8, for IHC).
